# National Alcohol Survey of households in Trinidad and Tobago (NASHTT): Alcohol use in households

**DOI:** 10.1186/s12889-017-4266-z

**Published:** 2017-04-20

**Authors:** R.G. Maharaj, M.S. Motilal, T. Babwah, P. Nunes, R. Brathwaite, G. Legall, S.D. Reid, M.E. Canavan, E.H. Bradley

**Affiliations:** 1 Unit of Public Health and Primary Care, Department of Paraclinical Sciences, The University of the West Indies, St. Augustine, Trinidad; 2 Unit of Psychiatry, Department of Medicine, The University of the West Indies, St. Augustine, Trinidad; 3 The Healthy Caribbean Coalition (HCC), St. Michael, Barbados; 4 Global Health Leadership Institute, Yale University, New Haven, CT USA

**Keywords:** Alcohol use, Caribbean, Intra-household relationships, Employment problems

## Abstract

**Background:**

To determine the patterns of alcohol use among households in Trinidad and Tobago (T&T) and to estimate the association between alcohol use and negative psychological, social, or physical events experienced by the household.

**Methods:**

A convenience sample of 1837 households across T&T. We identified bivariate correlates of alcohol use, and heavy episodic drinking using chi-square and t-test analyses and used multivariable logistic regression to estimate adjusted associations between household alcohol use and experiences within the past 12 months adjusted for sociodemographic covariates.

**Results:**

One thousand five hundred two households had complete data for all variables (82% response rate). Nearly two thirds (64%) of households included alcohol users; 57% of household that consumed alcohol also reported heavy episodic drinking. Households that reported alcohol consumption were significantly more likely to report illnesses within the households, relationship problems, and behavioral and antisocial problems with children. Among households where a member was employed, those who consumed alcohol were nearly twice as likely (OR = 1.98; 95% confidence interval (CI) 1.03, 3.82) to have a household member call in sick to work and 2.9 times as likely (OR = 2.9; CI 1.19, 7.04) to have a household member suffer work related problems compared with households who reported not consuming alcohol.

**Conclusions:**

Approximately two thirds of households in T&T reported using alcohol. These households were more likely to report psychological, physical, and social problems. These findings would support efforts to enforce current policies, laws, and regulations as well as new strategies to reduce the impact of harmful alcohol consumption on households in T&T.

**Electronic supplementary material:**

The online version of this article (doi:10.1186/s12889-017-4266-z) contains supplementary material, which is available to authorized users.

## Background

Although the literature is replete with evidence about high alcohol use in Trinidad and Tobago (T&T) [1–7], previous studies have focused only on the use and consequences of use for individuals and have neglected the potential impact of alcohol use on the larger household in which the users reside. Findings from the extant literature indicate moderate per capita consumption of alcohol (6.7 L per capita) in T&T but high rates of heavy episodic drinking (HED) or binge drinking (20.4% of population and 39.9% of drinkers, respectively) [8]. In 2015, males from T&T have been recorded as having the highest frequency of HED in the Americas in the past 30 days [9]. Although legal restrictions on sales of alcohol to minors exist, they are poorly enforced, and 84% of adolescents in T&T report having used alcohol previously [1], 25% of 13–15 year olds report having become drunk at some point in their lives [10], and 31% of university students report binge drinking [11].

The effects of alcohol are not only on the users [12], but also can be experienced by the family as use may affect relationships within the household. Nevertheless, we could find no studies that examined the effect of alcohol on the household, including influence on intra-household relationships and employment for adult members of the household. We used the term household to mean the person or group of persons who co-reside in, or occupy a dwelling [13]. The norms established in the household may influence the initiation of use among other members of the household, including children and youth [1]. Additionally there are impacts on the work place and employment, the roadways and in relationships with friends and acquaintances [14–16].

Accordingly, this study sought to quantify the prevalence of alcohol use by household and identify the correlates and household consequences of such alcohol use, with particular attention to intra-household relationships and employment. We used a large face-to-face survey to document both alcohol use and various household-level events in T&T. Findings from this study may be useful for supporting policies and practices to curb alcohol consumption that may contribute to negative household outcomes in T&T.

## Methods

### Study design and sampling procedure

**Fig. 1:**
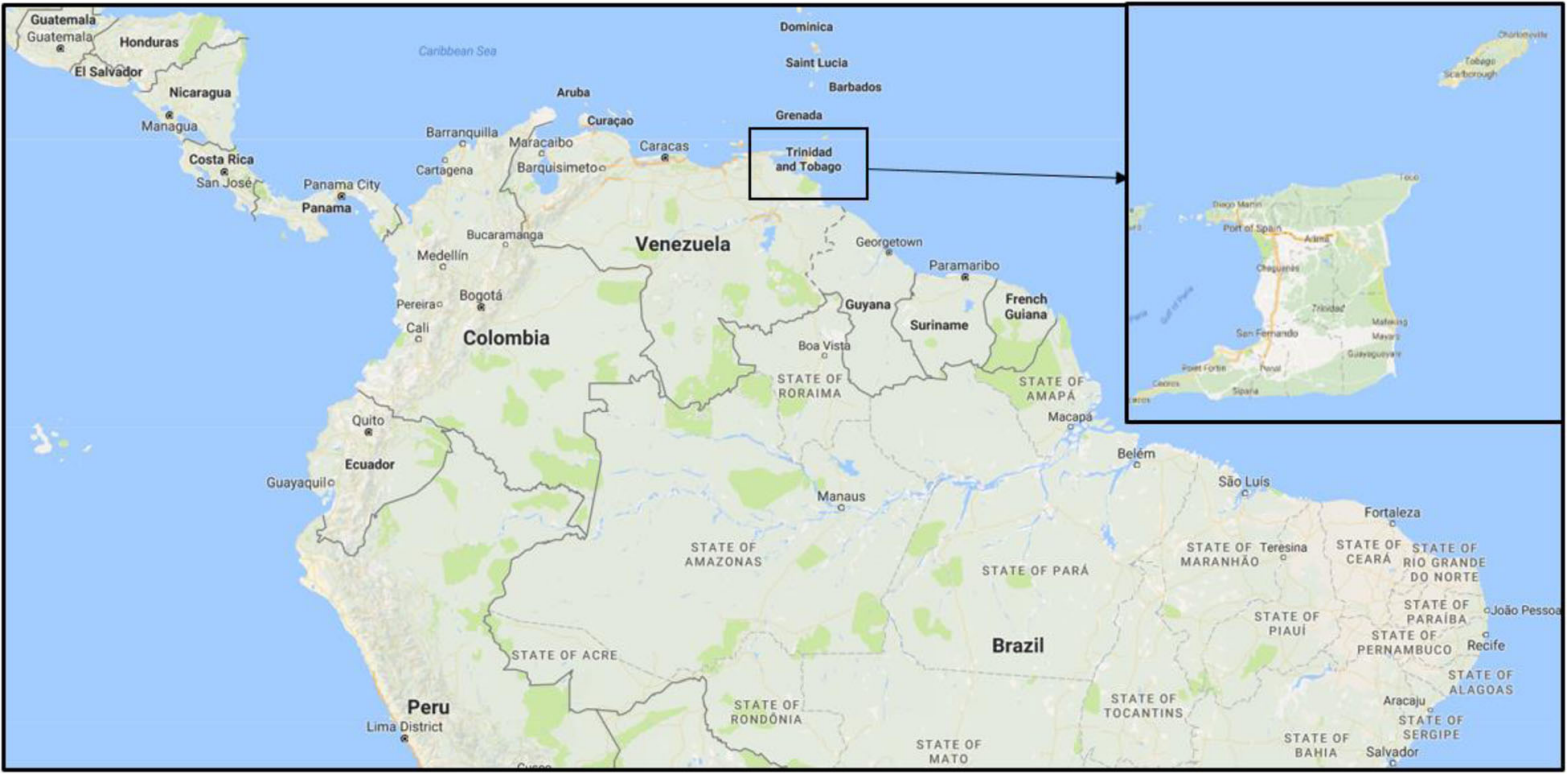
Map of Trinidad and Tobago (Source: Map data ©2017 Google)

We conducted a cross-sectional study using a sample of 1837 households that were approached across both Trinidad and Tobago islands (Fig. 1). The Central Statistical Office of T&T provided a list of 2824 national Enumeration Districts (EDs), and from these we drew a random sample of EDs. EDs represent geographically defined set of households with similar economic status. Using a random number generator, 53 EDs were selected and maps obtained for each. Each ED map had specific starting and end points to ensure that all households within each ED had an opportunity to be included in the final sample. Using the ED maps, interviewers visited an average of 35 households per ED using a systematic sampling interval of 3–6 households for each ED. Larger EDs (greater than 300 households) used a sampling interval of 6, medium EDs (between 250 and 300 households) used a sampling interval of 5, and small EDs (less than 250 households) used a sampling interval of 3. To choose the pattern when entering areas with multiple household such as apartments, enumerators started with the building to the left and began with the first apartment on the ground floor working in the sampling interval for each specific ED and following the sequence from bottom to top of the building. In cases in which a household refused to participate (*n* = 142 households, yielding a response rate of 92%, or 1695 out of 1837 households approached), the next successive household was approached until a response was attained. After each response, the sampling interval for that ED was reapplied. In the case that the household on the map was found to be a condominium or apartment building, the same interval strategy was used, counting each condominium or apartment as a household.

We selected the survey respondent within the household by asking the person who answered the door to identify the household head and interviewed that person. If the household head could not be contacted, we next selected the person aged 18 years or older who was most knowledgeable of the household. Only one questionnaire per household was allowed, but more than one person could contribute to answers to reflect the overall household experience. Information on the study was shared after the household respondent was identified. The interviewer read out the preamble to the questionnaire which provided extensive information on the purpose of the study and what would be required of participants. Agreeing participants completed and signed the accompanying consent form. Participants were given their signed consent form and signed a separate consent sheet for researchers’ records. The project was approved by the Ethics Committee of the Faculty of Medical Sciences, The University of the West Indies, St. Augustine, Trinidad.

### Survey instrument

A de novo questionnaire was created after reviewing the literature for relevant items and was constructed through a consultative process with family physicians, a psychiatrist, a statistician and public health specialists. Documents such as the Ministry of Health of T & T’s Draft National Policy on Alcohol and Babor’s *Alcohol: No Ordinary Commodity* [17] provided background information for the instrument. The survey instrument contained 50 items altogether and was developed over the period January 2012 through March 2013. The instrument is included in the Additional file 1 **.**

All data were collected in English by face-to-face interviews by experienced interviewers from the Central Statistical Office. A training manual and field manual were created and the interviewers underwent a half-day training session. Pre-testing of the instruments was carried out through first review by a sociologist for cultural context and then with the interviewers in a workshop setting for feasibility. The interviewers then pre-tested on individuals from the population for flow of language, comprehension of the questions by respondents, and time required to complete the questionnaire.

### Measures

#### Independent variables

Our primary independent variable was household alcohol consumption. Households were classified as consuming alcohol if at least 1 male or female adult in the household was reported to consume any alcoholic beverages within the past year. We also evaluated the frequency of heavy episodic drinking (HED). HED was defined as having at least 1 household member consuming 6 or more drinks in one sitting within the past 30 days.

#### Dependent variables

The main dependent variables were household experiences. We reported 12 general household experiences and 4 work related household experiences. Respondents indicated whether or not each of the following experiences had happened to one or more members of the household within the past 12 months: falling sick; having a lifestyle related illness (major illness e.g., heart, kidney, liver); experiencing relationship problems between partners (arguing, not talking); having strained relationships within the household; separation between spouses or partners; infidelity or cheating between spouses or partners; behavioral problems in children at home; antisocial problems of children at home/school; violent behavior; verbal abuse; police intervention in household dispute; or receiving a traffic ticket. Additionally, as a subgroup analysis among those participants whose household had at least one person employed, respondents were asked if at least one person in their household had experienced any of the following events within the past 12 months: calling in sick to work; suffering work related problems; experiencing a loss of job due to non-attendance at work; or loss of job due to work related problems.

#### Covariates

We evaluated the association between alcohol consumption and household experiences adjusted for several sociodemographic covariates. Self-reported major ethnicity of the household was classified as African, East Indian or Mixed/Other. Self-reported monthly income was assessed using 3 categories: low income, low middle income, or upper middle income/high income. Education level achieved by the head of household was assessed as primary school or less, secondary school, trade/vocation school, or university or tertiary level. Dwelling type was defined as private house, private apartment, Housing Development Authority housing, or part of a commercial or other building type. Employment was defined as having at least 1 household member who reported having full time or part time employment. Among households that consumed any alcohol within the past year, respondents were asked to note the households’ viewpoint regarding alcohol consumption: alcohol consumption is a normal part of the households daily life; alcohol consumption at family gatherings is a normal occurrence; in this household, we discuss the dangers of alcohol use, drinking alcohol is considered a pleasurable activity, alcohol is consumed in the presence of children, young people are allowed to drink alcohol in preparation for adult life and alcohol is a reward for a hard day’s work; taking a drink together helps ease household tensions; sometimes a personal and/or household problems are resolved over a drink; drinking together enables the household members to cope with many of the pressures of life; alcohol consumption should not be allowed to affect household relations; and if any of the members of the household drink against the household wishes.

### Statistical analysis

We used standard descriptive statistics to characterize the study population and reported alcohol use within the past year at the household level. We conducted chi-square statistics to identify bivariate correlates of alcohol use, and heavy episodic drinking. Multivariable logistic regression models were used to estimate and test the statistical significance of associations between household alcohol use and household experiences within the past 12 months adjusted for the following sociodemographic covariates: major ethnicity of the household, household income, highest level of schooling completed by the head of household, and household dwelling type. Subgroup analysis examined the association between household alcohol use within the past year and employment related problems among households with at least one member employed and the association between heavy episodic drinking and household experiences among household that reported any drinking. All analyses accounted for the complex survey design using household level clustered standard errors and were performed using SAS software, version 9.2 (SAS institute, Cary, NC).

## Results

### Description of the sample

Of the 1837 households approached, 1502 households had complete data for alcohol consumption within the past year, sociodemographic factors, and household experiences (response rate 82%). Within the sample, 954 households (64% of responding households) reported that at least 1 adult consumed alcohol within the past year. Additionally, among the 954 households that reported any alcohol consumption within the past year, 797 households also responded to the question of heavy episodic drinking (15% of the 954 households that reported alcohol use within the past year did not respond to this question and were excluded from the sub group analysis). Of the 797 households that answered the HED question, 57% (*n* = 457) reported heavy episodic drinking.

### Alcohol use and its association with socioeconomic factors and household experiences

Several sociodemographic factors and household experiences were significantly associated with household alcohol consumption within the past year **(**Table 1 **)**. In unadjusted analysis, alcohol consumption was significantly associated with reported monthly income, education level and dwelling type as well as with all 12 of the general household experiences (Table 1). In multivariable analysis adjusted for the sociodemographic covariates, we found that households that reported at least 1 member of the household consumed alcohol were significantly more likely to report relationship problems including infidelity, violent behavior, verbal abuse, and behavioral and antisocial problems with children (*p*-values <0.05). We found no statistical difference in household members’ sickness or illness and police interventions in household disputes based on alcohol consumption **(**Table 2 **).**

**Table 1:** Household characteristics and events by alcohol consumption status (*N* = 1502)

	No alcohol N (%)	Uses alcohol N (%)	*P* value
	(*n* = 548)	(*n* = 954)	
Household sociodemographic factors			
Major ethnicity of the household			0.074
African	249 (45.4)	380 (39.8)	
East Indian	157 (28.7)	284 (29.8)	
Mixed/other	142 (25.9)	290 (30.4)	
Reported monthly income			<0.001
Low income	202 (36.9)	251 (26.3)	
Low middle income	253 (46.2)	551 (57.8)	
Upper middle income/high income	93 (17.0)	152 (15.9)	
Education level achieved by head of household			<0.001
Primary School or less	203 (37.0)	264 (27.7)	
Secondary School	184 (33.6)	428 (44.9)	
Trade / Vocational	23 (4.2)	58 (6.1)	
University Tertiary	138 (25.2)	204 (21.4)	
Dwelling type			<0.001
Private House	86 (15.7)	162 (17.0)	
Private Apartment	188 (34.3)	246 (25.8)	
Part of commercial building/other	171 (31.2)	292 (30.6)	
Housing Development Authority	103 (18.8)	254 (26.6)	
Household experiences in last 12 months			
Household member falling sick	110 (20.1)	257 (26.9)	0.003
Lifestyle related illness (major illness e.g., heart, kidney, liver)	70 (12.8)	168 (17.6)	0.014
Relationship problems between partners (arguing, not talking)	17 (3.1)	133 (13.9)	<0.001
Strained relationships within household	5 (0.9)	81 (8.5)	<0.001
Separation between spouses or partners	1 (0.2)	16 (1.7)	0.009
Infidelity or cheating between spouses or partners	1 (0.2)	22 (2.3)	0.001
Behavioral problems in children at home	7 (1.3)	41 (4.3)	0.001
Antisocial problems of children at home/school	1 (0.2)	26 (2.7)	<0.001
Violent behavior	2 (0.4)	17 (1.8)	0.016
Verbal abuse	7 (1.3)	39 (4.1)	0.002
Police Intervention in household dispute	2 (0.4)	13 (1.4)	0.102
Receiving a traffic ticket	4 (0.7)	37 (3.9)	<0.001

**Table 2:** Adjusted^a^ odds ratio of household experiences for households reporting alcohol consumption compared with households reporting no alcohol consumption (*N* = 1502)

Household Experiences in last 12 months	Adjusted Odds Ratio	95% Confidence Interval	*p*-value
Household members falling sick	1.5	(0.9, 2.4)	0.137
Lifestyle related illness (major illness e.g., heart, kidney, liver)	1.4	(1.0, 2.0)	0.091
Relationship problems between partners (arguing, not talking)	4.4	(2.4, 8.0)	<0.001
Strained relationships within household	9.3	(3.9, 22.2)	<0.001
Separation between spouses or partners	9.1	(1.0, 82.7)	0.049
Infidelity or cheating between spouses or partners	12.3	(1.5, 102.5)	0.021
Behavioral problems in children at home	2.7	(1.0, 7.6)	0.054
Antisocial problems of children at home/school	12.5	(1.7, 93.3)	0.014
Violent behavior	4.6	(1.0, 21.3)	0.049
Verbal abuse	3.0	(1.4, 6.6)	0.007
Police Intervention in household dispute	3.6	(0.7, 19.1)	0.131
Receiving a traffic ticket	5.0	(1.3, 18.6)	0.018

^a^Models are adjusted for reported household major ethnicity, income category, highest level of schooling completed by the head of household, and dwelling type

### Alcohol use and its association with household experiences among employed

In secondary analysis using data from households that reported having at least 1 individual employed (*n* = 1349), we found households that consumed alcohol were nearly twice as likely (OR = 1.98; 95% confidence interval (CI) 1.03, 3.82) to have a household member call in sick to work and 2.9 times more likely (OR = 2.9; 95% CI 1.19, 7.04) to have a household member suffer work related problems compared with households who reported not consuming alcohol, adjusted for sociodemographic covariates **(**Table 3 **).** We found no statistically significant association between reported alcohol use and job loss due to work non-attendance or work related problems (*P*-values 0.247 and 0.095).

**Table 3:** Unadjusted and adjusted^a^ odds ratios of households reporting employment-related problems by households reporting alcohol consumption compared with households reporting no alcohol consumption (*N* = 1349 households in which at least one member was employed)

Household experiences in last 12 months	Unadjusted odds ratio (95% CI)	*p*-value	Adjusted odds ratio (95% CI)	*p*-value
Household member called in sick to work	2.35 (1.21, 4.59)	0.012	1.98 (1.03, 3.82)	0.042
Household member suffered work related problems	3.06 (1.23, 7.60)	0.016	2.90 (1.19, 7.04)	0.019
Household member experienced loss of job due to non-attendance at work	2.77 (0.59, 13.05)	0.199	2.58 (0.52, 12.84)	0.247
Household member experienced loss of job due to work related problems	3.53 (0.77, 16. 81)	0.104	3.71 (0.80, 17.26)	0.095

^a^Models are adjusted for household major ethnicity, income category, highest level of schooling completed by the head of household, and dwelling type

### Heavy episodic drinking and its association with socioeconomic factors and experiences

In subgroup analysis among households that responded to the frequency of heavy episodic drinking within the past 30 days (*n* = 797), we observed significant variation in sociodemographic factors and household experiences based on heavy episodic drinking status among households that reported alcohol use **(**Table 4 **)**. In unadjusted analysis, heavy episodic drinking was significantly associated with major ethnicity of the household, reported monthly income, education level and dwelling type as well as with the following general household experiences: household members’ sickness or illness, relationship problems between partners, strained relationships within the household, behavioral problems in children at home, and receipt of a traffic ticket (Table 4). Furthermore, in unadjusted analysis, heavy episodic drinking was significantly associated with the following viewpoints: alcohol consumption is a normal part of the households daily life; alcohol consumption at family gatherings is a normal occurrence; in this household, drinking alcohol is considered a pleasurable activity, alcohol is consumed in the presence of children, young people are allowed to drink alcohol in preparation for adult life and alcohol is a reward for a hard day’s work; taking a drink together helps ease household tensions; sometimes a personal and/or household problems are resolved over a drink; and drinking together enables the household members to cope with many of the pressures of life **(**Table 5 **)**.

**Table 4:** Distribution of sociodemographic variables and experiences among households by heavy episodic drinking (HED) within the past 30 days status among households that reported alcohol consumption (*N* = 797)

	No HED N (%)	HED N (%)	*P* value
	(*n* = 347)	(*n* = 450)	
Household sociodemographic factors			
Major ethnicity of the household			<0.001
African	158 (45.5)	139 (30.9)	
East Indian	95 (27.4)	156 (34.7)	
Mixed/other	94 (27.1)	155 (34.4)	
Reported monthly income			0.002
Low income	106 (30.6)	96 (21.3)	
Low middle income	196 (56.5)	263 (58.4)	
Upper middle income/high income	45 (13.0)	91 (20.2)	
Education level achieved by head of household			<0.001
Primary School or less	96 (27.7)	117 (26.0)	
Secondary School	164 (47.3)	203 (45.1)	
Trade/Vocational	28 (8.1)	14 (3.1)	
University Tertiary	59 (17.0)	116 (25.8)	
Dwelling type			0.013
Private House	70 (20.2)	69 (15.3)	
Private Apartment	101 (29.1)	105 (23.3)	
Part of commercial building/other	98 (28.2)	136 (30.2)	
NHA/HDC	78 (22.5)	140 (31.1)	
Household experiences in last 12 months			
Household members falling sick	85 (24.5)	148 (32.9)	0.012
Lifestyle related illness (major illness e.g., heart, kidney, liver)	50 (14.4)	100 (22.2)	0.006
Relationship problems between partners (arguing, not talking)	31 (8.9)	85 (18.9)	<0.001
Strained relationships within household	19 (5.5)	52 (11.6)	0.003
Separation between spouses or partners	5 (1.4)	10 (2.2)	0.601
Infidelity or cheating between spouses or partners	3 (0.9)	13 (2.9)	0.071
Behavioral problems in children at home	7 (2.0)	30 (6.7)	0.002
Antisocial problems of children at home/school	6 (1.7)	18 (4.0)	0.093
Violent behavior	5 (1.4)	11 (2.4)	0.446
Verbal abuse	11 (3.2)	25 (5.6)	0.123
Police Intervention in household dispute	4 (1.2)	8 (1.8)	0.567
Receiving a traffic ticket	7 (2.0)	28 (6.2)	0.005

**Table 5:** Associations between respondent’s viewpoint and heavy episodic drinking (HED) within the past 30 days among households that reported alcohol consumption (*N* = 797)

Respondent’s viewpoint	No HED N (%) (*n* = 347)	HED N (%) (*n* = 450)	*P*-value
Alcohol consumption is a normal part of this HH daily life	34 (9.8)	100 (22.2)	<0.001
Alcohol consumption at family gatherings is a normal occurrence	228 (65.7)	344 (76.4)	0.002
In this household, we discuss the dangers of alcohol use	189 (54.5)	254 (56.4)	0.759
In this household, drinking alcohol is considered a pleasurable activity	101 (29.1)	306 (68.0)	<0.001
In this household, alcohol is consumed in the presence of children	82 (23.6)	157 (34.9)	0.013
In this household, alcohol is a reward for a hard day’s work	44 (12.7)	163 (36.2)	<0.001
Taking a drink together helps ease household tensions	41 (11.8)	143 (31.8)	<0.001
Sometimes a personal and/or household problems are resolved over a drink	33 (9.5)	129 (28.7)	<0.001
Drinking together enables the household members to cope with many of the pressures of life	27 (7.8)	129 (28.7)	<0.001
In this household, young people are allowed to drink alcohol in preparation for adult life	39 (11.2)	111 (24.7)	0.001
Alcohol consumption should not be allowed to affect household relations	210 (60.5)	258 (57.3)	0.634
Do any of the members of the household drink against the household wishes	37 (10.7)	57 (12.7)	0.580

In fully adjusted multivariable models, households who reported heavy episodic drinking were more likely to have a household member with a lifestyle related illness (OR = 1.6; 95% CI 1.0, 2.4), and more likely to report relationship problems with partners (OR = 2.2; 95% CI 1.1,4.4), strained relationships within the household (OR = 2.1; 95% CI 1.1, 4.1), infidelity (OR = 4.1; 95% CI 1.2, 13.9), behavioral problems in children (OR = 3.1; 95% CI 1.0, 9.6), and receiving a traffic ticket (OR = 2.7; 95% CI 1.2, 5.9) **(**Table 6 **)**.

**Table 6:** Adjusted^a^ odds ratio of household experiences for households reporting heavy episodic drinking compared with households reporting no heavy episodic drinking within the past 30 days among households that reported alcohol consumption (*N* = 797)

Household experiences in last 12 months	Adjusted Odds Ratio	95% Confidence Interval	*p*-value
Household members falling sick	1.3	(0.9, 2.0)	0.182
Lifestyle related illness (major illness e.g., heart, kidney, liver)	1.6	(1.0,2.4)	0.043
Relationship problems between partners (arguing, not talking)	2.2	(1.1, 4.4)	0.022
Strained relationships within household	2.1	(1.1, 4.1)	0.032
Separation between spouses or partners	1.9	(0.5, 7.2)	0.367
Infidelity or cheating between spouses or partners	4.1	(1.2, 13.9)	0.023
Behavioral problems in children at home	3.1	(1.0, 9.6)	0.048
Antisocial problems of children at home/school	2.3	(0.9, 6.1)	0.101
Violent behavior	1.7	(0.6, 4.9)	0.312
Verbal abuse	1.8	(0.9, 3.7)	0.101
Police Intervention in household dispute	1.6	(0.5, 5.3)	0.406
Receiving a traffic ticket	2.7	(1.2, 5.9)	0.015

^a^Models are adjusted for major ethnicity of the household, household income category, highest level of schooling completed by the head of household, and household dwelling type

## Discussion

We found that almost two thirds of households in T&T reported that at least one member consumed alcohol and of these, nearly half reported heavy episodic drinking in the last month. Compared with households that reported not consuming alcohol, households that reported consuming alcohol were significantly more likely to report acute and chronic illnesses, intra-household difficulties (including arguments between partners and infidelity), problems with children, violence and abuse, and job related difficulties among those who were employed. Heavy episodic drinking also elevated risk of negative experiences including illnesses within the household, relationship problems between partners, strained relationships within the household, behavioral problems in children at home, and receipt of traffic tickets. Importantly, households with alcohol use generally had higher income and education, suggesting that these issues are prominent among otherwise advantaged households in T&T. Although higher income has been associated with alcohol use in Australia [18], many studies have shown excessive alcohol consumption to be more prevalent among people with lower education and income [19, 20].

The prevalence of heavy episodic drinking reported is cause for concern as ill effects of heavy episodic drinking have well been documented on overall health, executive functioning, stroke and total mortality [21–24]. Although uncommon, the other negative experiences of work related problems, depression, being involved in a motor vehicle accident, financial problems and behavioral problems of children both in home and school are still worthy of mention because of the impact these can have on the society. Most troublesome was the normalization of alcohol use reported by respondent from households where heavy episodic drinking was reported in the last month. More than two-thirds of respondents from households with heavy episodic drinking indicated that alcohol use was a pleasurable activity and a normal part of family gatherings; only 58% of these households had discussed the dangers of alcohol use.

Implications of the findings are several. The data suggest ample opportunity for public education about the effects of alcohol use, particularly heavy episodic drinking. These effects include not only personal impact but also negative experiences for the household. Furthermore, the negative effects cover a wide range of health, interpersonal, and employment consequences, which may be avoidable with reductions in alcohol use. Because alcohol use part of everyday life in T&T and an important part of both the culture and the economy, efforts to reduce its consumption will require careful strategy and time. School based programs have been shown to be successful in some settings, and systematic reviews concluded that several interventions can be successful in reducing widespread alcohol use [25, 26]. This literature suggests strong evidence for policies on alcohol sale and taxation; mixed evidence for family, community, school and mass media interventions; and only weak evidence for workplace and illicit sale interventions. Although designing and implementing effective, evidence-based interventions will be challenging, our data suggest addressing this issue could have substantial benefits in T&T, in terms of health improvements, better intra-household relationships, and fewer work-related problems for households.

Although our results provide the first documentation of which we are aware for an English speaking Caribbean country, the findings should be interpreted in light of several limitations. First, the data were self-reported and it is possible that particularly negative experiences have been under-reported due to social desirability bias. We anticipate that such bias would be more likely among those reporting higher alcohol use, so we believe this would be a bias toward the null resulting if anything in an underestimate of the associations we reported. Second, the data are cross-sectional and therefore cannot be used to infer causality; nevertheless, as a first study of alcohol use and household experiences in T&T, the data are helpful and suggest future longitudinal cohort studies are warranted. Last, we conducted this study in T&T, and hence the data may not be generalizable to other countries, although it nonetheless provides context for countries of like geography and culture and may spawn collaborative efforts in the Caribbean and neighboring areas to conduct similar studies.

## Conclusion

We found that alcohol use was prominent in T&T and households with members that use alcohol are at elevated risk for multiple negative consequences including worse health, challenging intra-household relationships, and employment problems. Furthermore, households with heavy episodic drinking are at even greater risk, despite the frequency with which such alcohol consumption is normalized as part of social gatherings and everyday household life. These findings offer opportunities to approach changes in culture and family education, to remove the widespread acceptance of alcohol as a family activity, and to encourage safer alcohol use.

## Additional file

Additional file 1: National Household Alcohol Survey. (PDF 269 kb)
