# Supplementary material for: National Alcohol Survey of households in Trinidad and Tobago (NASHTT): Alcohol use in households
Source: BMC Public Health. 2017 Apr 20;17:347. doi: 10.1186/s12889-017-4266-z (PMC5399432; doi:10.1186/s12889-017-4266-z)
Supplement: Additional file 1: — National Household Alcohol Survey. (PDF 269 kb) [file 12889_2017_4266_MOESM1_ESM.pdf]

NATIONAL HOUSEHOLD ALCOHOL SURVEY

Thank you for taking the time to answer this questionnaire. This survey has 5 parts and should take approximately 25 minutes to complete. The objectives of the study are:

- ***a. To determine the extent of alcohol use among households in Trinidad and Tobago***
- ***b. To determine the impact of alcohol on the psychological and social fabric of households in Trinidad and Tobago***
- ***c. To determine the changes to alcohol regulations, advertisements and other policies desired by households in Trinidad and Tobago***

In the first section, you will be asked about your household's use of alcohol. In part 2, you will be asked about challenges to the psychological and social fabric of your household in the past 12 months. Questions in part 3 focus on your observation of the impact of places that sell alcohol in your community. Then we would like you to indicate whether you agree with certain proposed changes in legislation and policies with regards to alcohol consumption, advertising and sale of alcohol in Trinidad and Tobago. In the final section, we will be grateful if you provide some brief details about your household. If there are any questions you are uncomfortable with you do not have to answer them. All information will be confidential and your name will not be attached to any information shared about this study. We thank you in advance for your time.

OFFICIAL USE ONLY

Date administered: dd/mm/yyyy

Community area: __________________ ED No: _______________ HH No.________________

Respondent is

A. The head of household [Yes] [No]

B. Over 18 years of age and knowledgeable about the household [Yes] [No]

C. Only one (1) questionnaire per household but more than one person contributed /participated at times [Yes] [No]

Time survey began: _______________

Time survey ended: _______________

Name of enumerator: __________________ Signature: ___________________________

NATIONAL HOUSEHOLD SURVEY OF ALCOHOL AND ITS IMPACT ON THE HOUSEHOLD

PART 1: HOUSEHOLD'S USE OF ALCOHOL

1. How many persons live in this household?

   Adult Males _____ Adult Females _____ Children less than 18yrs _____

2. How many adults in this household consume alcohol? Males ______ Females _____

   [If no one in the household consumes alcohol, ***Skip to Q4 and 5***]

3. Where do members of this household consume alcohol, select as many as apply?

   [ ] At home [ ] At bars or rum shops [ ] On beach limes/excursions

   [ ] At social gatherings [ ] At wakes/funerals; etc. [ ] Other *(Specify)* _____________

4. What are the reasons that no one in this household consumes alcohol, select as many as apply?

   [ ] Family agreement [ ] Tenant Agreement [ ] Health issues

   [ ] Religious beliefs/practices [ ] Other reasons *(Specify)* ________________________________

5. Has anyone in this household ever mentioned being (or feeling) pressured to consume alcohol at parties, events or when liming with friends? [ ] Yes [ ] No

   [If no one in the household consumes alcohol, *Skip to Part 2*]

6. Which of the following statements represents your household experience or viewpoint on alcohol (select as many as applies):

   a) Alcohol consumption is a normal part of this household's daily life [ ]
   b) Alcohol consumption at family gatherings is a normal occurrence [ ]
   c) Taking a drink together helps ease household tensions [ ]
   d) Drinking together enables the household members to cope with many of the pressures of life [ ]
   e) Sometimes personal and/or household problems are resolved over a drink [ ]
   f) In this household drinking alcohol is considered a pleasurable activity [ ]
   g) In this household alcohol is a reward for a hard day's work [ ]
   h) In this household young people are allowed to drink alcohol in preparation for adult life [ ]
   i) In this household we discuss the dangers of alcohol use [ ]
   j) Alcohol consumption should not be allowed to affect household relations [ ]
   k) In this household alcohol is consumed in the presence of children [ ]
   l) What are some other beliefs this household has regarding alcohol use?

   ____

   ____

m) Do any members of the household drink against the household's wishes? Yes [ ] No [ ]

Now I am going to ask some questions about the use of alcoholic beverages in this household <u>during this past year</u>. Some local examples of alcoholic beverages are Carib or Stag beers; Stout e.g. Mackeson or Guinness; Malt liquor drinks e.g. Smirnoff Ice; Rum e.g. Black Label, Vat 19; Wine; vodka; tequila and other spirits. A standard drink (10g of alcohol) may be a can or a bottle of beer; a glass of wine or about 5 teaspoons of spirits or alcohol without a chaser.

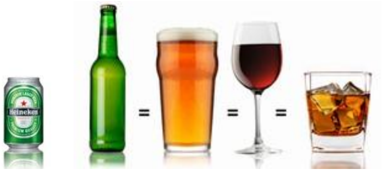

7. How many days a week do adults in this household who consume alcohol, do so at home?
   [ ] Everyday [ ] 0 days [ ] 1-2 days [ ] 3-4 days [ ] 5-6 days

8. How many days a week do adults in this household who consume alcohol do so while away from home?
   [ ] Everyday [ ] 0 days [ ] 1-2 days [ ] 3-4 days [ ] 5-6 days

9. Do the adults in this household who drink alcohol consume more alcohol away from home than at home?
   [ ] Mostly at home [ ] Mostly when away from home [ ] Equally often

10. How many of each of the following drinks would you say is normally consumed by the **adult** in this household who drinks the least and who drinks the most, **per session**?

**Items**	**Drinks the least**	**Drinks the most**
1. Cans/ Glass or bottle of beer/stout	[ ]	[ ]
2. Drink of rum/scotch/vodka	[ ]	[ ]
3. Glasses of wine	[ ]	[ ]
4. Other, list…..	[ ]	[ ]

11. Many persons in T&T drink heavily, how often does anyone in this household have six or more drinks on one occasion?
    [ ] Daily or almost every day [ ] Weekly [ ] Fortnightly
    [ ] Monthly [ ] Never [ ] Don't know

12. Many persons are unable to stop once they start to drink, how many members of this household are usually unable to stop drinking once started?
    Number of males: __________ Number of females: _______

13. We know many young persons have access to alcohol, how many persons in this household, less than 18 yrs, do you suspect drink alcohol?

    Number of males: _________ Number of females: ______

14. Many young persons drink in school, how many persons in this household, less than 18 years have been suspended from school because of alcohol? Number of males: _________ Number of females: ______

15. What are some reasons why the adults in this household drink?

    [ ] To get going in the morning [ ] For pleasure [ ] To escape emotional pain

    [ ] To feel good/function better [ ] Cannot help it [ ] To escape physical pain

    [ ] Other reason: ____

16. How much money would you say the adults in this household who drink spend per week on drinks?

    [ ] Less than TT$100 [ ] $1- $200 [ ] $300-$400 [ ] $500 or more

17. Think of the heaviest drinker in this household, how regularly **per week** do they get drunk?

a.	At Home:	[ ] 0	[ ] 1-2	[ ] 3-4	[ ] 5-7	[ ] Weekends only
b.	Away from home:	[ ] 0	[ ] 1-2	[ ] 3-4	[ ] 5-7	[ ] Weekends only

PART 2: CHALLENGES TO THE SOCIAL FABRIC OF THE HOUSEHOLD IN THE PAST 12 MONTHS

Which of the following has at least one member of this household experienced **in the past twelve (12) months**? **PLEASE NOTE THAT THIS SECTION DOES NOT APPLY SPECIFICALLY TO ALCOHOL.**

**EXPERIENCE**	**Past 12 months**	**No**
18. Self neglect (not bathing, eating, grooming)		
19. Relationship problems between partners (arguing, not talking)		
20. Strained relationships within the household		
21. Lack of money for basic household needs/Financial hardship (cannot buy school books, food, diapers)		
22. Disconnection of utilities (phone, electricity, water, cable etc)		
23. Loss of property due to failure to pay mortgage, loans, rent or bills		
24. Household members falling sick (minor illness)		

**EXPERIENCE**	**Past 12 months**	**No**
25. Lifestyle-related illness (major illness e.g. heart, kidney, liver etc)		
26. Household members calling in sick to work		
27. Work related problems (e.g. Always late for work or making errors on the job)		
28. Loss of job due to non-attendance at work		
29. Loss of job due to work related problems		
30. Receiving a traffic ticket (e.g. exceeding the speed limit)		
31. Been involved in a vehicular accident		
32. Injury to a family member in a vehicular accident in which a family member was the driver.		
33. Death to a family member in a vehicular accident in which a family member was the driver.		
34. Injury to a family member in a vehicular accident in which the other driver was drunk or under the influence of alcohol		
35. Death to a family member in a vehicular accident in which the other driver was drunk or under the influence of alcohol		
36. Infidelity or cheating between spouses or partners		
37. Separation between spouses or partners		
38. Death of a member (other than through a vehicle accident)		
39. Financial problems		
40. Being diagnosed or suffering with depression		
41. Having to seek counselling (e.g. from pastor, priest, imam, pundit or psychologist)		
42. Attempting suicide		
43. Committing suicide		
44. Abuse between partners in the household		
45. Abuse leading to having a restraining order taken out		
46. Police intervention in household disputes		
47. Abuse resulting in admission to the hospital or emergency room		

**EXPERIENCE**	**Past 12 months**	**No**
48. Behavioural problems of children at home		
49. Behavioural problems of children at school (truancy, disregard authority/ teacher)		
50. Anti-social problems of children at home/school (fighting, bullying)		
51. Learning problems of children (decreased performance or always failing)		
52. Risky sexual behaviour (e.g. sex with a non-regular partner; unprotected sex)		
53. Unwanted pregnancy		
54. Partner abuse		
55. Verbal abuse		
56. Violent behaviour		
57. Sexual abuse		

58. Has any of the above **EVER happened (i.e. greater than 12 months ago)** to a member of this household? If 'Yes", please list the item's number above:

____

PART 3: HOUSEHOLD'S OPINION OF ALCOHOL TRADE AND THE RESIDENTIAL ENVIRONMENT

These questions will ask your response on any bars or rum shops operating in your community.

59. How many bars or places where alcohol is purchased are within walking distance of your household?

[ ] 0 [ ] 1-3 [ ] more than 3

60. Is anyone in your household been annoyed by the number of bars or rum shops operating within in your community? [ ] No [ ] Yes [ ] DK

61. Would anyone in your household like to see fewer bars or rum shops operating in your community?

[ ] No [ ] Yes [ ] DK

62. Is anyone in your household disturbed by the noise emerging from the bars or rum shops?

[ ] No [ ] Yes [ ] DK

63. Is anyone in your household disturbed by the patrons using the bars or rum shops in your community?

[ ] No [ ] Yes [ ] DK

Part 4: TACKLING ALCOHOL ISSUES IN A NATIONAL CAMPAIGN

These questions will ask about your household's willingness to support changes, through signing a petition or casting a vote, regarding national alcohol policies in Trinidad and Tobago.

64. Which of the following changes regarding alcohol do you believe members of this household would support in a national campaign? **TICK as many as the household is willing to support. If <u>not</u> willing to support please <u>leave blank.</u>**

    a. Set the legal age for drinking at 21 years [ ]
    b. Restricting alcohol advertisements on TV/radio/newspapers/cinema [ ]
    c. Ban of all alcohol advertisements on TV/radio/newspapers/cinema [ ]
    a. Ban of all alcohol advertisements at cultural or sporting events. [ ]
    b. Delinking the consumption of alcohol with social or sexual success [ ]
    c. Delinking the consumption of alcohol with driving or physical performance [ ]
    d. Ban of radio stations playing songs which reference alcohol use [ ]
    e. Stricter and more intensive enforcement of breathalyzer [ ]
    f. Holding sellers of alcohol responsible for the amount of alcohol they sell to patrons. [ ]
    g. Advocating for proof of age to be shown before alcohol is sold to a buyer [ ]
    h. More prominent warning labels on products displaying the alcohol concentration [ ]
    i. More prominent warning labels on products displaying the harmful effects of alcohol [ ]
    j. Increased taxation on alcohol [ ]
    k. Increase the public education campaigns on responsible alcohol use in all settings including in schools [ ]
    l. Increased fines for drunk driving [ ]
    m. Reduction in the opening hours of bars and rum shops. [ ]

    [if item 'm' selected go to question below on opening hours of bars and rum shops]

62. During what days and times do you feel bars or rums shops should be allowed to open?
    1. Any day, any time [ ]
    2. Specific days ___________________________________
    3. Specific times ___________________________________

63. Respondents' comments on this survey

    ____

    ____

PART 5: TELL US ABOUT YOUR HOUSEHOLD

Finally we would like you please tell us about your household.

64. What is the major ethnicity of this household?

    [ ] African [ ] East Indian [ ] Mixed [ ] Other

65. Number of persons employed full time_______________

66. Number of persons employed part time_______________

67. In what category would you place your household?

    [ ] Low income [ ] Low Middle Income [ ] Upper Middle Income [ ] High Income

68. Type of dwelling

    (1) Separate private house (2) NHA/HDC house (3) Private apartment

    (4) NHA/HDC townhouse (5) Private townhouse (6) Part of commercial building

    (7) NHA/HDC apartment (8) Other________________________

69. Main structure of outside walls of dwelling

    (1) Brick or concrete (2) Wood (3) Wood, brick and concrete

    (4) Wood and galvanize (5) Wattle, Adobe or Tapia (6) Boxboard or plyboard

    (7) Other______________

70. Highest level of schooling completed by the head of household

    (1) None (2) Primary school (3) Post-primary or school-leaving

    (4) Secondary School (5) Trade/vocational (6) University or Tertiary level

71. Highest educational qualification attained by the head of household

    (1) None (2) School leaving certificate (3) CXC/O'Level certificate

    (4) CAPE/ GCE A' levels Certificate (5) Diploma/Associate degree

    (6) Undergraduate Degree (e.g. BA, BSc) (7) Postgraduate Degree (e.g. Masters, PhD.)

72. In which **monthly** income bracket would you say this household falls?

    (1) Less than $1000 (2) between $1000-1999

    (3) Between $2,000- $4, 999 (4) Between $5,000 -$7,999

    (5) Between $8,000-$9,999 (6) Between $10, 000- $19,999

    (7) $20, 000-$29,999 (8) Greater than $30 000

THE END.

PLEASE THANK THE RESPONDENT(S) AND HOUSEHOLD!!

PLEASE LEAVE THIS PAGE WITH THE RESPONDENT/HOUSEHOLD

Do you or someone you know have a special story about how alcohol has affected your life or that of someone in this household and would like to share that story? If yes, you are invited to contact the Principal Investigator, Dr. Rohan Maharaj at 770 6953 or our secretary at 645-6741 to schedule an interview. Or give your name and telephone number to the interviewer. Please keep these separate from the survey form or the consent form.
